# Polarization control with an X-ray phase retarder for high-time-resolution pump–probe experiments at SACLA^1^

**DOI:** 10.1107/S1600577519006222

**Published:** 2019-06-20

**Authors:** Y. Kubota, M. Suzuki, T. Katayama, K. Yamamoto, K. Tono, Y. Inubushi, T. Seki, K. Takanashi, H. Wadati, M. Yabashi

**Affiliations:** a Japan Synchrotron Radiation Research Institute (JASRI), 1-1-1 Kouto, Sayo-cho, Sayo-gun, Hyogo 679-5198, Japan; b RIKEN SPring-8 Center, 1-1-1 Kouto, Sayo-cho, Sayo-gun, Hyogo 679-5148, Japan; c Institute for Solid State Physics, The University of Tokyo, 5-1-5 Kashiwanoha, Kashiwa, Chiba 277-8581, Japan; d Institute for Materials Research, Tohoku University, 2-1-1 Katahira, Aoba-ku, Sendai, Miyagi 980-8577, Japan; e Center for Spintronics Research Network, Tohoku University, 2-1-1 Katahira, Aoba-ku, Sendai, Miyagi 980-8577, Japan

**Keywords:** X-ray free-electron lasers, polarization, dynamical theory of diffraction

## Abstract

Control of the polarization of an X-ray free-electron laser (XFEL) has been performed using an X-ray phase retarder (XPR) in combination with an arrival timing diagnostic on BL3 of the SPring-8 Angstrom Compact free-electron LAser (SACLA). To combine with the timing diagnostic, a pink beam was incident on the XPR crystal and then monochromated in the vicinity of samples. A high degree of circular polarization of ∼97% was obtained experimentally at 11.567 keV, which agreed with calculations based on the dynamical theory of X-ray diffraction. This system enables pump–probe experiments to be operated using circular polarization with a time resolution of 40 fs to investigate ultrafast magnetic phenomena.

## Abstract 2

Polarization control using an X-ray phase retarder in combination with an arrival timing diagnostic on BL3 of SACLA is reported.

## 1. Introduction   

X-ray free-electron lasers (XFELs) based on the self-amplified spontaneous emission (SASE) principle (Kondratenko & Saldin, 1980 ▸; Bonifacio *et al.*, 1984 ▸) produce brilliant femtosecond light pulses with ultrahigh coherence in the X-ray region (Emma *et al.*, 2010 ▸). The SPring-8 Angstrom Compact free-electron LAser (SACLA), constructed in Harima, Japan, is an XFEL facility with three beamlines: BL2 and BL3 for hard XFEL beams (Ishikawa *et al.*, 2012 ▸; Yabashi *et al.*, 2015 ▸), and BL1 for a soft XFEL beam (Owada, Togawa *et al.*, 2018 ▸). Their pulse durations were evaluated to be 7.7 fs on BL3 (Inubushi *et al.*, 2012 ▸, 2017 ▸) and below 100 fs on BL1 (Kubota *et al.*, 2019 ▸; Owada *et al.*, 2019 ▸). Using arrival timing diagnostic systems between the XFEL and synchronized optical laser pulses, the time resolution in pump–probe experiments has been improved to less than 100 fs (Katayama *et al.*, 2016 ▸; Owada, Nakajima *et al.*, 2018 ▸; Owada *et al.*, 2019 ▸).

Polarization is a fundamental property of light. In the X-ray region, anisotropic and magnetic properties of matter have been widely investigated using various polarization states. A diamond X-ray phase retarder (XPR) (Hirano *et al.*, 1991 ▸; Giles *et al.*, 1994 ▸; Lang & Srajer, 1995 ▸) has been installed on BL3 of SACLA to control the polarization of the hard XFEL beam (Suzuki *et al.*, 2014 ▸) so as to study the ultrafast dynamics of chemical bonding and magnetic states. In particular, pump–probe X-ray magnetic circular dichroism (XMCD) spectroscopy is a powerful method to investigate spin dynamics such as a demagnetization and magnetization reversal observed on a picosecond to femtosecond time scale (Kirilyuk *et al.*, 2010 ▸; Takubo *et al.*, 2017 ▸). Since the diamond XPR crystal has its diffraction plane rotated by 45° from the horizontal, the horizontally polarized incident XFEL beam is decomposed into equal parts σ- and π-polarized radiation relative to the diffraction plane. According to the dynamical theory of X-ray diffraction (Batterman & Cole, 1964 ▸), the XPR crystal produces a phase retardation between these two components near the Bragg condition, and consequently one is able to control the polarization states. In a conventional configuration [which we call a ‘mono-XPR’ (or scheme A) configuration], a monochromatic beam with a bandwidth of Δ*E*/*E* ≃ 1 × 10^−4^ produced with a perfect crystal monochromator is employed as the incident beam on the XPR crystal, because an energy bandwidth and/or angular divergence can cause degradation of the polarization states converted with the XPR crystal (Suzuki *et al.*, 2014 ▸). However, one may consider locating the XPR crystal upstream of the monochromator [*i.e.* an ‘XPR-mono’ (or scheme B) configuration] so as to expand the applicable range of experiments with various polarization states. For example, scheme B enables us to combine the XPR system with the arrival timing monitor (TM) on BL3 of SACLA, because the TM system requires the transport of a pink beam with a bandwidth of Δ*E*/*E* ≃ 5 × 10^−3^ through the beamline optics (including the XPR crystal) located in the optics hutch (OH) (Katayama *et al.*, 2016 ▸).

In this study, we evaluated the degree of circular polarization (*P*_C_) of the XFEL beam in scheme B that combines polarization control with the TM on BL3 of SACLA. We experimentally confirmed a high degree of *P*_C_, which is consistent with the calculated value and almost the same as that for scheme A (Suzuki *et al.*, 2014 ▸).

## 2. Experimental   

**Figure 1:**
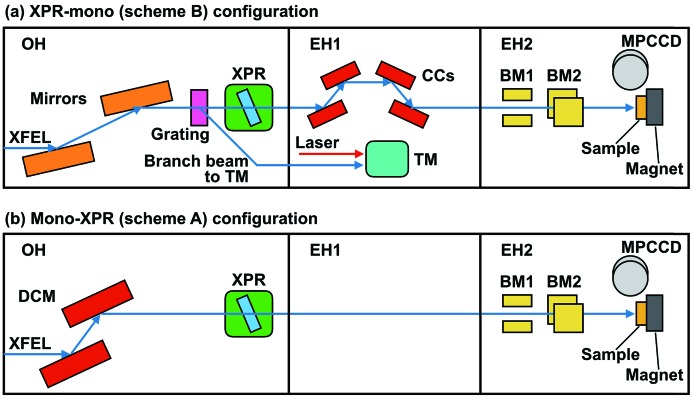
Schematic drawings of the experimental setup of (*a*) the XPR-mono (scheme B) configuration and (*b*) the mono-XPR (scheme A) configuration (see the main text) on BL3 of SACLA. XPR: X-ray phase retarder; TM: timing monitor; CCs: monochromator composed of channel-cut crystals; BM: beam monitor; MPCCD: multiport charge-coupled device; DCM: double-crystal monochromator.

Fig. 1(*a*) ▸ shows the experimental setup with the XPR-mono (scheme B) configuration on BL3 of SACLA (Tono *et al.*, 2013 ▸). In the OH, the pink XFEL beam reflected with mirrors was incident on the XPR crystal (Suzuki *et al.*, 2014 ▸). In this study, a diamond (100) crystal 1.5 mm thick was used in a 220 symmetric Laue geometry. A branch beam of the XFEL, separated with a grating in the OH, was used for the arrival TM in experimental hutch EH1 (Katayama *et al.*, 2016 ▸). After passing through the XPR crystal, the main XFEL beam was monochromated at the Pt *L*_3_ edge with a pair of channel-cut crystals (CCs) in a (+, −, −, +) geometry, which maintains the height of the beam, with Si(111) reflections installed in EH1.

For comparison, we set up the conventional mono-XPR (scheme A) configuration as shown in Fig. 1 ▸(*b*). A double-crystal monochromator (DCM) with Si(111) reflections was used to produce the monochromatic beam. The monochromatic XFEL pulse was incident on the XPR crystal. The grating, TM and CCs monochromator were not used in this configuration.

In EH2, we installed two Kapton scattering beam monitors (BMs) to measure the intensities of the horizontal and vertical linear polarized components as shown in Fig. 1 ▸. An FePtPd film (50 nm thick) grown on an MgO(100) substrate by magnetron sputtering at 773 K was used to evaluate the *P*_C_ of the XFEL by measuring the XMCD. The sample forms an *L*1_0_ structure with an out-of-plane easy magnetization direction (Seki *et al.*, 2011 ▸; Takubo *et al.*, 2017 ▸). Using an Nd–Fe–B permanent magnet, an external magnetic field of μ_0_ *H* = ±0.59 T was applied along the surface normal of the sample film to saturate the magnetization. The sample was maintained at room temperature during measurements. We used two pairs of the sample and the permanent magnet, with opposite field directions for each pair, to measure the XMCD signal. The two samples were cut from the same parent film, and the magnetic field strengths were prepared to be nearly the same for the two configurations. The remaining systematic errors in the magnetic asymmetry were corrected. The XFEL beam was incident on the sample in the surface normal direction. To measure the absorbed intensity at the Pt *L*_3_ edge, we detected the Pt *L*_α_ (9.443 keV) fluorescence with a multiport charge-coupled device (MPCCD) detector (Kameshima *et al.*, 2014 ▸), as shown in Fig. 1 ▸.

## 3. Results and discussion   

**Figure 2:**
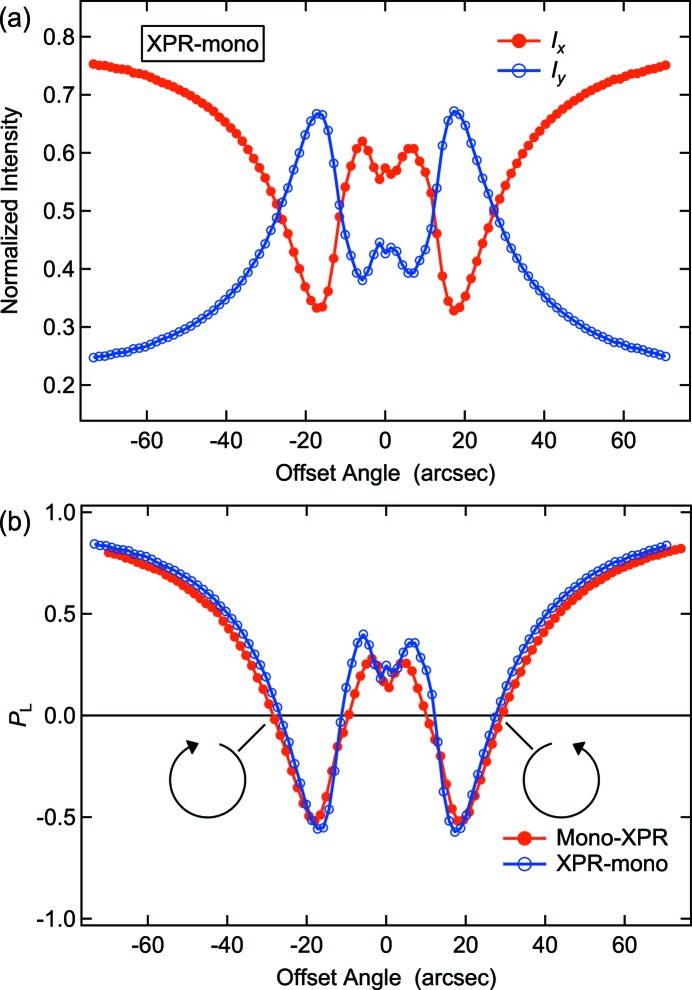
(*a*) The intensities of the horizontal (*I*_*x*_, red solid circles) and vertical (*I*_*y*_, blue open circles) polarization components as a function of the offset angle of the XPR crystal in the XPR-mono (scheme B) configuration at 11.567 keV. (*b*) The XPR crystal angle dependence of *P*_L_. The red solid circles and blue open circles represent the values of *P*_L_ in the mono-XPR (scheme A) and XPR-mono (scheme B) configurations, respectively.

Fig. 2 ▸(*a*) shows the intensities of the horizontal (*I*_*x*_) and vertical (*I*_*y*_) polarization components as a function of the offset angle of the XPR crystal from the 220 symmetric Laue geometry at 11.567 keV detected with the Kapton scattering BMs in scheme B. This photon energy was selected so as to maximize the value of the XMCD. From the measured values of *I*_*x*_ and *I*_*y*_, the degree of linear polarization, *P*_L_, was determined using the equation
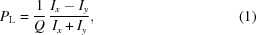
where *Q* is the correction factor of the BMs used in this experiment. The factor was estimated to be *Q* = 0.5990 ± 0.0005 by a measurement of the intensity of the horizontally polarized component without the XPR crystal (Suzuki *et al.*, 2014 ▸). Fig. 2 ▸(*b*) shows the XPR crystal angle dependence of *P*_L_. We also operated the same measurement in scheme A. One finds good agreement between the two configurations, which shows that polarization control with the XPR crystal works properly even for scheme B. At the points of *P*_L_ = 0, the XPR generates ±π/2 phase retardation, and we obtain circular polarization with right- and left-helicity, respectively. A small discrepancy between the two configurations is due to the difference between the bandwidths obtained with the CCs and DCM (Suzuki *et al.*, 2014 ▸). The monochromatic beam generated with the CCs has a bandwidth about 0.8 times smaller than that obtained with the DCM due to the difference in their reflection geometries: (+, −, −, +) for the CCs and (+, −) for the DCM.

**Figure 3:**
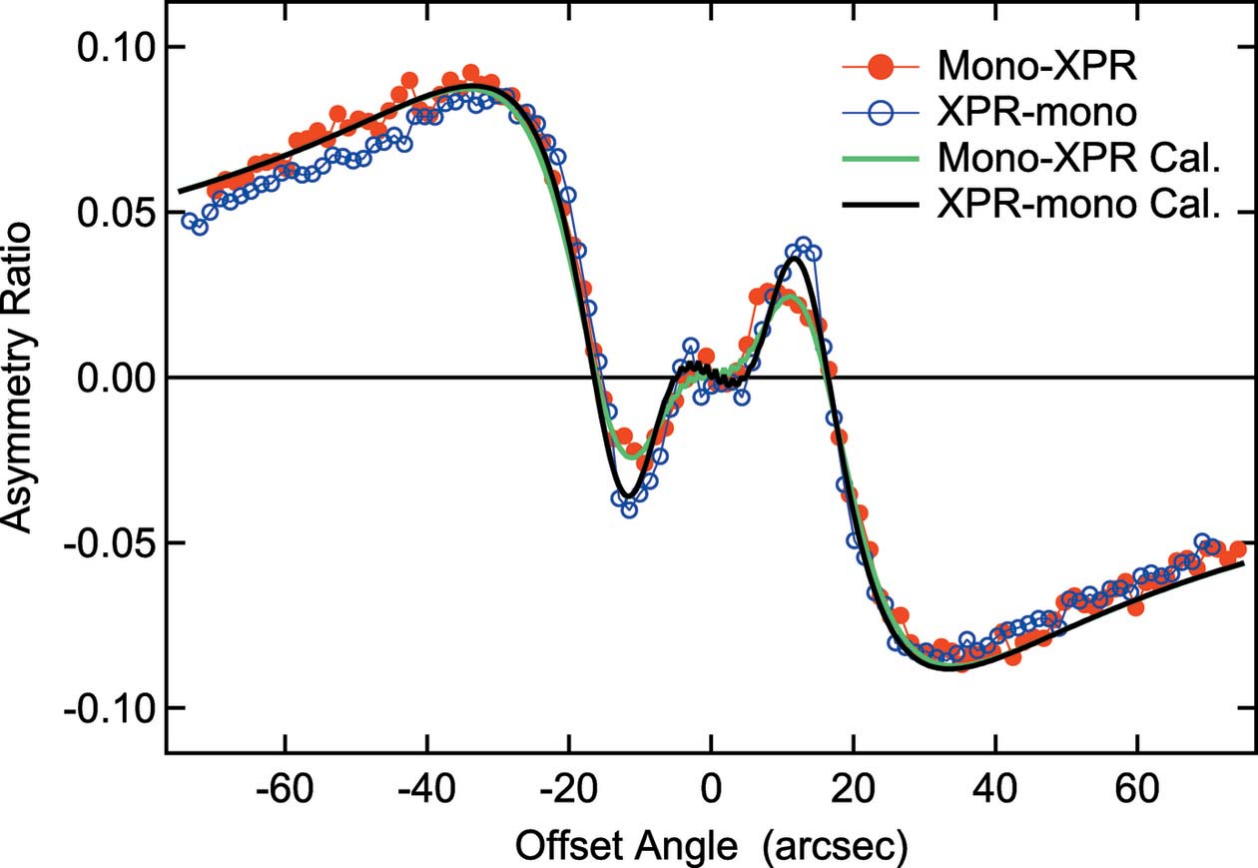
The magnetic asymmetry ratio obtained from the FePtPd film at the Pt *L*_3_ edge as a function of the offset angle of the XPR crystal. The red solid circles and blue open circles represent the values in the mono-XPR (scheme A) and XPR-mono (scheme B) configurations, respectively. The green and black solid curves represent the calculated values for the scheme A and B configurations assuming Gaussian bandwidths of Δ*E*/*E* = 1.1 × 10^−4^ and 8.6 × 10^−5^, respectively.

Next, we estimated *P*_C_ in scheme B by measuring the XMCD of the FePtPd film at the Pt *L*_3_ edge. A magnetic asymmetry ratio, *R*, was determined by the equation
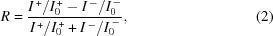
where 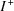 (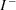) is the absorbed intensity in the FePtPd film detected with the MPCCD for a magnetic field applied in the direction antiparallel (parallel) to the X-ray incident direction. 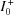 (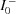) is the intensity of the incident XFEL beam, which is the sum of the output of the BMs. Fig. 3 ▸ shows the values of *R* as a function of the offset angle of the XPR crystal in schemes A and B, with the calculation curves obtained by the same method as that used in a previous study (Suzuki *et al.*, 2014 ▸). This result indicates that *P*_C_ in scheme B is almost the same as that of scheme A with a high value of 0.97, which was obtained in the previous study (Suzuki *et al.*, 2014 ▸). The uncertainty in *P*_C_ in scheme B was estimated to be ±0.06. The discrepancy in the offset angles between −20 and 20 arcsec is due to the influence of the bandwidth, which was seen in *P*_L_ (Suzuki *et al.*, 2014 ▸). For negative offset angles, *R* obtained in scheme B is lower than that in scheme A, which is probably due to a glitch as discussed in the previous study (Suzuki *et al.*, 2014 ▸).

**Figure 4:**
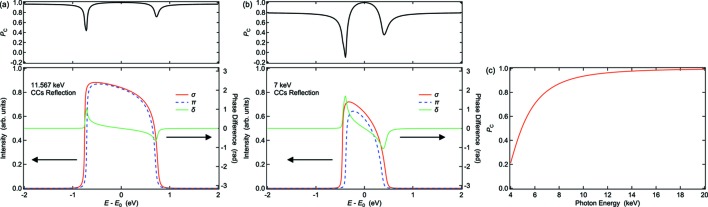
Calculated results based on the dynamical theory of X-ray diffraction. (*a*, *b*) The values of *P*_C_ (top), and rocking curves with the additional phase difference (δ) between the σ- and π-polarization components (bottom) after the four-fold reflections of the CCs monochromator as a function of relative photon energy *E* − *E*_0_ at (*a*) *E*_0_ = 11.567 keV and (*b*) *E*_0_ = 7 keV. The black solid lines represent the values of *P*_C_. The red solid and blue dashed lines represent rocking curves for the σ- and π-polarization components (left axes), respectively. The green solid lines represent the values of δ (right axes). (*c*) Calculated values of *P*_C_ after the four-fold reflections of the CCs monochromator as a function of photon energy. The values of *P*_C_ were obtained by weighted averaging.

From the experiments, we confirmed that a high degree of *P*_C_ was retained in scheme B at 11.567 keV. However, according to the dynamical theory of X-ray diffraction (Batterman & Cole, 1964 ▸), a CCs monochromator could introduce a phase difference (δ) between the σ- and π-polarization components, which leads to degradation of the polarization states produced with the XPR crystal. To investigate the effect of CCs on *P*_C_ over a wide photon energy range, we calculated the values of *P*_C_ based on the dynamical theory of diffraction. Since the angular divergence of the XFEL beam (∼2 µrad) is sufficiently smaller than the Darwin width of the Si(111) reflection (∼20 µrad), we only considered the effect of the energy spread. Fig. 4 ▸(*a*) shows rocking curves for the σ- and π-polarization components and the resulting δ and *P*_C_ after the four-fold reflections of the CCs at *E*_0_ = 11.567 keV. For the calculation of *P*_C_, we assumed that the incident XFEL beam has circular polarization with *P*_C_ = 1. Although *P*_C_ decreased at the edges of the rocking curves, the weighted average value was maintained at 0.96 at 11.567 keV. This result is consistent with our experimental results, showing that *P*_C_ in scheme B has almost the same value as that in scheme A. Note that the experimental value of *P*_C_ ≃ 0.97 in scheme B included the effects of not only the degradation by the CCs but also the narrower bandwidth than that obtained by the DCM. Fig. 4 ▸(*c*) shows the weight-averaged values of *P*_C_ after the four-fold reflections of the CCs as a function of photon energy. This result shows that a high *P*_C_ can be maintained in a high photon energy region (larger than ∼10 keV), which includes XMCD for the *L* edges of 5*d* elements such as Pt, Ir and Os (Wienke *et al.*, 1991 ▸; Schütz *et al.*, 1989 ▸). On the other hand, the value of *P*_C_ decreases drastically in scheme B in a lower energy region of less than ∼10 keV, which includes the *K* edges of 3*d* transition metals and the *L* edges of rare earth elements (Schütz *et al.*, 1987 ▸, 1989 ▸; Giles *et al.*, 1994 ▸). For example, Fig. 4 ▸(*b*) shows rocking curves with δ and the values of *P*_C_ after the four-fold reflections of the CCs at *E*_0_ = 7 keV. To improve *P*_C_ in this energy region, a single CC monochromator can be used in scheme B. Alternatively, a high-intensity monochromatic beam generated with the self-seeded XFEL scheme (Amann *et al.*, 2012 ▸; Lindberg & Shvyd’ko, 2012 ▸; Inoue *et al.*, 2019 ▸) is available for ultrafast magnetic measurements.

## References

[bb1] Amann, J., Berg, W., Blank, V., Decker, F. J., Ding, Y., Emma, P., Feng, Y., Frisch, J., Fritz, D., Hastings, J., Huang, Z., Krzywinski, J., Lindberg, R., Loos, H., Lutman, A., Nuhn, H. D., Ratner, D., Rzepiela, J., Shu, D., Shvyd’ko, Y., Spampinati, S., Stoupin, S., Terentyev, S., Trakhtenberg, E., Walz, D., Welch, J., Wu, J., Zholents, A. & Zhu, D. (2012). *Nat. Photon.* **6**, 693–698.

[bb2] Batterman, B. W. & Cole, H. (1964). *Rev. Mod. Phys.* **36**, 681–717.

[bb3] Bonifacio, R., Pellegrini, C. & Narducci, L. M. (1984). *Opt. Commun.* **50**, 373–378.

[bb4] Emma, P., Akre, R., Arthur, J., Bionta, R., Bostedt, C., Bozek, J., Brachmann, A., Bucksbaum, P., Coffee, R., Decker, F., Ding, Y., Dowell, D., Edstrom, S., Fisher, A., Frisch, J., Gilevich, S., Hastings, J., Hays, G., Hering, P., Huang, Z., Iverson, R., Loos, H., Messerschmidt, M., Miahnahri, A., Moeller, S., Nuhn, H., Pile, G., Ratner, D., Rzepiela, J., Schultz, D., Smith, T., Stefan, P., Tompkins, H., Turner, J., Welch, J., White, W., Wu, J., Yocky, G. & Galayda, J. (2010). *Nat. Photon.* **4**, 641–647.

[bb5] Giles, C., Malgrange, C., Goulon, J., de Bergevin, F., Vettier, C., Fontaine, A., Dartyge, E. & Pizzini, S. (1994). *Nucl. Instrum. Methods Phys. Res. A*, **349**, 622–625.

[bb6] Hirano, K., Izumi, K., Ishikawa, T., Annaka, S. & Kikuta, S. (1991). *Jpn. J. Appl. Phys.* **30**, L407–L410.

[bb28] Inoue, I., Osaka, T., Hara, T., Tanaka, T., Inagaki, T., Fukui, T., Goto, S., Inubushi, Y., Kimura, H., Kinjo, R., Ohashi, H., Togawa, K., Tono, K., Yamaga, M., Tanaka, H., Ishikawa, T. & Yabashi, M. (2019). *Nat. Photon.* **13**, 319–322.

[bb8] Inubushi, Y., Inoue, I., Kim, J., Nishihara, A., Matsuyama, S., Yumoto, H., Koyama, T., Tono, K., Ohashi, H., Yamauchi, K. & Yabashi, M. (2017). *Appl. Sci.* **7**, 584. 10.1038/s41598-018-35611-0 PMC6262013 30487583

[bb7] Inubushi, Y., Tono, K., Togashi, T., Sato, T., Hatsui, T., Kameshima, T., Togawa, K., Hara, T., Tanaka, T., Tanaka, H., Ishikawa, T. & Yabashi, M. (2012). *Phys. Rev. Lett.* **109**, 144801. 10.1103/PhysRevLett.109.144801 23083249

[bb9] Ishikawa, T., Aoyagi, H., Asaka, T., Asano, Y., Azumi, N., Bizen, T., Ego, H., Fukami, K., Fukui, T., Furukawa, Y., Goto, S., Hanaki, H., Hara, T., Hasegawa, T., Hatsui, T., Higashiya, A., Hirono, T., Hosoda, N., Ishii, M., Inagaki, T., Inubushi, Y., Itoga, T., Joti, Y., Kago, M., Kameshima, T., Kimura, H., Kirihara, Y., Kiyomichi, A., Kobayashi, T., Kondo, C., Kudo, T., Maesaka, H., Maréchal, X. M., Masuda, T., Matsubara, S., Matsumoto, T., Matsushita, T., Matsui, S., Nagasono, M., Nariyama, N., Ohashi, H., Ohata, T., Ohshima, T., Ono, S., Otake, Y., Saji, C., Sakurai, T., Sato, T., Sawada, K., Seike, T., Shirasawa, K., Sugimoto, T., Suzuki, S., Takahashi, S., Takebe, H., Takeshita, K., Tamasaku, K., Tanaka, H., Tanaka, R., Tanaka, T., Togashi, T., Togawa, K., Tokuhisa, A., Tomizawa, H., Tono, K., Wu, S., Yabashi, M., Yamaga, M., Yamashita, A., Yanagida, K., Zhang, C., Shintake, T., Kitamura, H. & Kumagai, N. (2012). *Nat. Photon.* **6**, 540–544.

[bb10] Kameshima, T., Ono, S., Kudo, T., Ozaki, K., Kirihara, Y., Kobayashi, K., Inubushi, Y., Yabashi, M., Horigome, T., Holland, A., Holland, K., Burt, D., Murao, H. & Hatsui, T. (2014). *Rev. Sci. Instrum.* **85**, 033110. 10.1063/1.4867668 24689567

[bb11] Katayama, T., Owada, S., Togashi, T., Ogawa, K., Karvinen, P., Vartiainen, I., Eronen, A., David, C., Sato, T., Nakajima, K., Joti, Y., Yumoto, H., Ohashi, H. & Yabashi, M. (2016). *Struct. Dyn.* **3**, 034301. 10.1063/1.4939655 PMC4733081 26958586

[bb12] Kirilyuk, A., Kimel, A. V. & Rasing, T. (2010). *Rev. Mod. Phys.* **82**, 2731–2784.

[bb13] Kondratenko, A. M. & Saldin, E. L. (1980). *Particle Accel.* **10**, 207–216.

[bb14] Kubota, Y., Inoue, I., Togawa, K., Kinjo, R., Iwayama, H., Harries, J. R., Inubushi, Y., Owada, S., Tono, K., Tanaka, T., Hara, T. & Yabashi, M. (2018). *16th International Conference on Megagauss Magnetic Field Generation and Related Topics (MEGAGAUSS 2018)*, 25–29 September 2018, Kashiwa, Japan (https://doi.org/10.1109/MEGAGAUSS.2018.8722659).

[bb15] Lang, J. C. & Srajer, G. (1995). *Rev. Sci. Instrum.* **66**, 1540–1542.

[bb16] Lindberg, R. R. & Shvyd’ko, Y. V. (2012). *Phys. Rev. ST Accel. Beams*, **15**, 050706.

[bb19] Owada, S., Fushitani, M., Matsuda, A., Fujise, H., Sasaki, Y., Hikosaka, Y., Hishikawa, A. & Yabashi, M. (2019). In preparation.

[bb17] Owada, S., Nakajima, K., Togashi, T., Kayatama, T. & Yabashi, M. (2018). *J. Synchrotron Rad.* **25**, 68–71. 10.1107/S1600577517015284 29271753

[bb18] Owada, S., Togawa, K., Inagaki, T., Hara, T., Tanaka, T., Joti, Y., Koyama, T., Nakajima, K., Ohashi, H., Senba, Y., Togashi, T., Tono, K., Yamaga, M., Yumoto, H., Yabashi, M., Tanaka, H. & Ishikawa, T. (2018). *J. Synchrotron Rad.* **25**, 282–288. 10.1107/S1600577517015685 PMC5741133 29271777

[bb21] Schütz, G., Frahm, R., Wienke, R., Wilhelm, W., Wagner, W. & Kienle, P. (1989). *Rev. Sci. Instrum.* **60**, 1661–1665. 10.1103/PhysRevLett.62.2620 10040038

[bb20] Schütz, G., Wagner, W., Wilhelm, W., Kienle, P., Zeller, R., Frahm, R. & Materlik, G. (1987). *Phys. Rev. Lett.* **58**, 737–740. 10.1103/PhysRevLett.58.737 10035022

[bb22] Seki, T., Iwama, H., Shima, T. & Takanashi, K. (2011). *J. Phys. D Appl. Phys.* **44**, 335001.

[bb23] Suzuki, M., Inubushi, Y., Yabashi, M. & Ishikawa, T. (2014). *J. Synchrotron Rad.* **21**, 466–472. 10.1107/S1600577514004780 24763633

[bb24] Takubo, K., Yamamoto, K., Hirata, Y., Yokoyama, Y., Kubota, Y., Yamamoto, S., Yamamoto, S., Matsuda, I., Shin, S., Seki, T., Takanashi, K. & Wadati, H. (2017). *Appl. Phys. Lett.* **110**, 162401.

[bb25] Tono, K., Togashi, T., Inubushi, Y., Sato, T., Katayama, T., Ogawa, K., Ohashi, H., Kimura, H., Takahashi, S., Takeshita, K., Tomizawa, H., Goto, S., Ishikawa, T. & Yabashi, M. (2013). *New J. Phys.* **15**, 083035.

[bb26] Wienke, R., Schütz, G. & Ebert, H. (1991). *J. Appl. Phys.* **69**, 6147–6149.

[bb27] Yabashi, M., Tanaka, H. & Ishikawa, T. (2015). *J. Synchrotron Rad.* **22**, 477–484. 10.1107/S1600577515004658 PMC4416664 25931056

